# Adaptive feature selection using v-shaped binary particle swarm optimization

**DOI:** 10.1371/journal.pone.0173907

**Published:** 2017-03-30

**Authors:** Xuyang Teng, Hongbin Dong, Xiurong Zhou

**Affiliations:** 1 College of Computer Science and Technology, Harbin Engineering University, Harbin, Heilongjiang, China; 2 Internal Medicine, Heilongjiang Provincial Corps Hospital Chinese People’s Armed Police Forces, Harbin, Heilongjiang, China; Beihang University, CHINA

## Abstract

Feature selection is an important preprocessing method in machine learning and data mining. This process can be used not only to reduce the amount of data to be analyzed but also to build models with stronger interpretability based on fewer features. Traditional feature selection methods evaluate the dependency and redundancy of features separately, which leads to a lack of measurement of their combined effect. Moreover, a greedy search considers only the optimization of the current round and thus cannot be a global search. To evaluate the combined effect of different subsets in the entire feature space, an adaptive feature selection method based on V-shaped binary particle swarm optimization is proposed. In this method, the fitness function is constructed using the correlation information entropy. Feature subsets are regarded as individuals in a population, and the feature space is searched using V-shaped binary particle swarm optimization. The above procedure overcomes the hard constraint on the number of features, enables the combined evaluation of each subset as a whole, and improves the search ability of conventional binary particle swarm optimization. The proposed algorithm is an adaptive method with respect to the number of feature subsets. The experimental results show the advantages of optimizing the feature subsets using the V-shaped transfer function and confirm the effectiveness and efficiency of the feature subsets obtained under different classifiers.

## Introduction

The generation and accumulation of data in every walk of life is giving rise to new requirements for data mining and machine learning tasks. The question about to utilize these growing data scientifically and extract their valuable information has become a popular topic of current research. A professor at the University of Washington who is an expert in machine learning has noted that the key to success in machine learning tasks is the correct use of data features. Therefore, the critical first step in extracting valuable information is to determine the important features in large data sets.

Feature selection refers to the assessment of features or feature combinations using a specific evaluation function to obtain a lower-dimensional feature subset from the original feature set. The significance of feature selection is that it can be effectively used to address the curse of dimensionality. The learning process can be more efficient when data are modeled in terms of certain important features [1]. Moreover, the elimination of useless and redundant features makes the results of learning result interpretable. This reduction does not change the physical meaning of the features. For this reason, feature selection methods tend to be used when it is necessary to understand the potential meaning and original characteristics of data sets. In the process of feature selection, better and fewer features mean more flexibility, and the learned model will also represent more approximate results with regard to the original data. In recent years, feature selection has been widely applied to studies of social networks [2], intrusion detection [3] [4], bioinformation [5], image analysis [6], nature language processing [7], etc.

Research on feature selection is mainly focused on two aspects of the process [1]: the search strategy and the evaluation function. In the task of choosing the subject of features from the original set that contains the most valuable information, an attempt to traverse all possible subsets would be computationally infeasible and would encounter the problem of combinatorial explosion. To allow features to be selected more efficiently, several greedy search strategies are used in this work, such as forward search and backward search. However, a traditional greedy search can easily fall into local optima, which means that non-optimal subsets may be found. This search strategy is inapplicable for finding the globally optimal solution or an approximately optimal solution. With respect to subset evaluation functions, a variety of metrics can be used, such as distance-based metrics [8], [9], information-entropy-based metrics [10] [11] [12] [13], correlation-based metrics [14] and dependency-based metrics [15]. In many of these approaches, features or pairs of features are evaluated for their importance one by one; consequently, the correlations among different combinations of features are ignored. Therefore, it is of interest to study an evaluation function that can be used to rapidly evaluate the importance of each feature subset as a whole. Depending on the methods used to evaluate feature subsets, feature selection models can be divided into three categories [16]: filter models, wrapper models and embedded models. A filter model relies only on the intrinsic characteristic of the data for feature selection, without any specific guidance from learning algorithms, e.g., [17]. A wrapper model requires a pre-specified learning algorithm, and the performance of each subset based on the chosen learning algorithm is used as the measure for determining the final feature subset, e.g., [18]. An embedded model includes the feature selection method as part of the objective analysis of a learning algorithm, which is used as part of the training process for determining feature importance,e.g., [19]. Depending on the output of the feature selection process, these can be further divided into two types: ranking methods and subset selection methods. A ranking method is based on the degree of importance of each feature, which is then used to select a feature subset, where the number of features in the selected subset must be controlled manually. By contrast, a subset selection method outputs the subset with the best overall evaluation.

Because of the advantages of filter models, such as generation ability and computing capacity, various efficient filter measures have been developed for use as the evaluation function during feature selection. Battiti et al. presented a mutual information measure for evaluating the degree of dependency between features and class labels in [10]; the larger the mutual information measure is, the more important the corresponding feature is. The minimum-redundancy-maximum-relevance(mRMR) method was proposed by Peng et al. as a classical information-based feature selection method [12]. This method uses mutual information to calculate the correlation between feature and class and the redundancy between features. However, these ranking methods cannot determine the combined effect of all features in a subset. In 2004, Yu et al. proposed an Fast Correlation Based Filter(FCBF) method for measuring feature-feature and feature-class correlations by using symmetrical uncertainty in the context of information theory [20]. However, this method cannot handle feature redundancy. In [21] and [22], Sun et al. used the Banzhaf power index and Shapley value to evaluate the combined influence of the features in each feature subset as a whole; however, this method required a significant amount of time during the process of creating candidate feature subsets. As a representative method based on distance, Hu et al. proposed the soft fuzzy rough sets(SFS) method [9], which uses the neighborhood rough set for the evaluation, but this method is strongly influenced by neighborhood parameters and dependency. In [8], Dong reported research on an evolutionary algorithm for evaluating feature subsets; however, this method relied on measures of within-class and between-class distances, which resulted in a weak anti-noise ability. In [15], Liu et al. considered the overall change in the dependency of the subsets to propose a novel feature selection based on a dependency margin; however, this method had a high time complexity. ReliefF [23] is the classical ranking method of feature selection based on distance measurements, but it lacks the ability to measure the combined effects of features.

The concept of correlation information entropy comes from the field of multi-sensor data fusion. It is a measure of redundant information in a multi-sensor system. This technique was proposed by Wang et al [24]. for measuring overlapping and independent information in a multi-sensor system. The information on the correlation among multiple sensors is measured as a value on the closed interval of [0,1]. The greater the degree of independence among sensors is, the smaller is the degree of redundancy (overlap), and consequently, the greater is the correlation information entropy. In our previous work [25], we used a greedy search based on the correlation information entropy for feature selection, in a method termed CMFS−*η*. In this method, the most relevant feature that introduces the lowest redundancy is added to the candidate subset until the redundancy constraint *η* is reached. This method considers only the optimal set for the current round [1]. As an example of a forward search, suppose that f5 is better than f6 in the third round, and consequently, the candidate subset is {*f*_2_,*f*_4_,*f*_5_}; however, in the fourth round, it may be that {*f*_2_,*f*_4_,*f*_6_,*f*_8_} is better than any{*f*_2_,*f*_4_,*f*_5_,*f*_*i*_}. Therefore, the greedy algorithm cannot obtain the optimal set. Similar problems arise in a backward search and a two-way search. The greedy strategy cannot solve a problem in which the combination of weaker features with other features results in a stronger recognition ability. As we all know, the search speed of particle swarm optimization (PSO) is better than it of genetic algorithm and some other evolutionary algorithms, while run time is an important indicator in feature selection. Therefore, the PSO is more suitable for searching the feature space [26] [27] [28] [29].Variants of PSO have been developed and discussed in recent study from the above references. However, different parameters and evolutionary strategies of PSO have great influence on search ability. Liu et al. [30] investigated the effect of the inertia weight on the behavior of BPSO theoretically and empirically. Du et al. [31] illustrated the importance of some useful information from other neighbors rather than the best performer. In their study, particles are influenced by only several top individuals of the population sorted by performance. The redundant information of neighbors is controlled and the useful information is shared by particles in their PSO method. Furthermore, the transfer function is another important influence of binary particle swarm optimization (BPSO) which should be tested for different optimization problems [32].

In this paper, each dimension of features is regarded as a sensor, and these sensors are used to model the sensor information system and the feature information system. Then, the correlation information entropy is calculated to measure the combined effects of the feature subsets. To overcome the shortcomings of ranking methods as discussed above, we use BPSO to search the feature space. Based on an analysis of transfer functions, we determine that V-shaped binary particle swarm optimization (VBPSO) is most appropriate for evaluating the feature subsets. Unlike traditional information theory methods, in the proposed feature selection method using V-shaped binary particle swarm optimization (VPFS), subsets are formed adaptively to prevent the greedy search from easily falling into local optima. The proposed method is independent of any specific learning algorithm; therefore, it is a filter model. Moreover, a comprehensive set of experiments is conducted to demonstrate the effectiveness of the proposed method. Finally, the main contributions of this paper can be summarized as follows:

- VBPSO is used for the first time for a global search for feature selection.
- The transformation between a sensor information system and a feature information system is established.
- A metric called the correlation information entropy is employed to measure the combined effects of features.
- Four indicators of performance based on statistical tests, compression ratio and run time, rather than classification accuracy alone, are used to experimentally verify the validity of the proposed method and evaluate various methods for comparison.

## Materials and methods

### Feature selection

Throughout the paper, the following notations are used. The feature information system is defined as a triplet *S* = (*D*,*F*,*C*), where *D* = {*d*_1_,*d*_2_, …,*d*_*k*_} is the complete dataset consisting of a total of k instances, *F* = {*f*_1_,*f*_2_, …,*f*_*n*_} is the set of *n* features, and *C* = {*c*_1_,*c*_2_, …,*c*_*m*_} is the set of target classes. Every instance di has *n* features (although the value of one or more features may be null). The instances in dataset *D* can be divided into *m* target classes. Every instance has only one target class in single-label classification. The main goal of feature selection is to find the optimal feature subset *P*, which contains *p* features, to represent the original features (generally, *p*≪ *n* if the dimensionality of the search space is high). A basic requirement is that the subset *P* must provide acceptable classification accuracy. A higher-level requirement is that the performance of a learning algorithm should be enhanced by using the selected feature subset. The exhaustive search and evaluation of all possible subsets are infeasible because the search space is exponentially large (= 2^*n*^-1), meaning that the problem is *NP* complete.

The feature selection task usually consists of four components: feature subset generation, subset evaluation, satisfaction of a termination condition and result verification. Based on a given search strategy, the candidate feature subset *P*’ is first generated (feature subset generation). Then, each candidate feature subset is evaluated using a given evaluation function and compared with the previous best candidate feature subset (subset evaluation). If the current feature subset is superior, the previous best feature subset is replaced. This loop of generation and evaluation continues until a specified termination condition is satisfied. Then, the selected feature subsets must be validated using certain learning algorithms.

### Binary particle swarm optimization

Particle swarm optimization (PSO), which was inspired by studies of bird predation behavior, is an evolutionary algorithm developed by Kennedy and Eberhart [33]. Particles are used to optimize the solutions in the search space and to record the best location on the current path. Each particle considers its own current position and velocity and records its own optimal solution (optimal position), *pbest*. Then, it adjusts its current position according to the global optimal solution among the population, *gbest*. The specific updating of each particle is performed as shown in Eqs (1) and (2):

$$v_{h}^{t+1}=wv_{h}^{t}+c_{1}\times rand\left(pbest_{h}-x_{h}^{t}\right)+c_{2}\times rand\left(gbest-x_{h}^{t}\right)\;$$ (1)

$$x_{h}^{t+1}=x_{h}^{t}+v_{h}^{t+1}\;$$ (2)

where $v_{h}^{t}$ is the velocity of the hth particle in iteration *t*, *w* is the inertia coefficient, and is the position of the *h*th particle in iteration *t*. The acceleration coefficients *c*_1_ and *c*_2_ are nonnegative constants that control the influence of *pbest* and *gbest* on the search process. In formula (1), $wv_{h}^{t}$ represents the search capabilities of particles, whereas $c_{1}\times rand(pbest_{h}-x_{h}^{t})$ and $c_{2}\times rand(gbest-x_{h}^{t})$ represent the evolution of the particles themselves and the cooperation among particles, respectively.

The original PSO algorithm was developed for solving problems in a continuous space. Kennedy later adjusted the method used to update velocity and position and proposed binary particle swarm optimization (BPSO), which is suitable for solving discrete problems [34]. In this approach, the particles in the population can search in a binary space. That is to say, the position vectors of the particles are represented by values of 0 or 1. The most important component of the BPSO algorithm is the transfer function, which converts continuous velocity values into discrete positions. The velocity obtained using Eq (1) is transformed into a vector in the interval [0,1] by means of the sigmoid function *T*, as given in Eq (3):

$$T\left(v_{h}^{k}\left(t\right)\right)=\frac{1}{1+e^{-v_{h}^{k}\left(t\right)}}\;$$ (3)

where $v_{h}^{k}(t)$ is the velocity of the *h*th particle in iteration *t* for the *k*th dimension. Hence, the position of a particle is updated to its new value using the following Eq (4):

$$x_{h}^{k}\left(t+1\right)=\left\{\begin{array}{cc} 0 & if\;\text{rand}<T\left(v_{h}^{k}\left(t+1\right)\right)\\ 1 & if\;\text{rand}\geq T\left(v_{h}^{k}\left(t+1\right)\right) \end{array}\right.\;$$ (4)

### Correlation information entropy

Let *P* represents the output sequences of a multi-sensor system with *n* sensors at time *t* (*t* = 1, 2, …, *m*),as defined in Eq (5):

$$P={\left(y_{i}\left(t\right)\right)}_{1\leq t\leq m,1\leq i\leq n},\;P\in R{}^{m\times n}\;$$ (5)

where *y*_*i*_(*t*) denotes the output of the *i*th sensor at time *t*. The correlation matrix at time *t* is generated using *P* and is described as in Eq (6):

$$R=P\cdot P^{T},\;R\in R{}^{n\times n}\;$$ (6)

In the correlation matrix, each entry indicates the similarity of the information between the two corresponding sensors and implies the overlap of information in the multi-sensor system. In practical applications, because the signals obtained from different sensors vary with their different ranges, the sensor data must be centralized and normalized before the correlation matrix can be calculated. The correlation matrix *R* of *n* sensors is derived as in Eq (7):

$$R=\left[\begin{array}{cccc} \begin{array}{c} 1\\ r_{21}\\ \vdots \\ r_{n1} \end{array} & \begin{array}{c} r_{12}\\ 1\\ \vdots \\ r_{n2} \end{array} & \begin{array}{c} \ldots \\ \ldots \\ \ldots \end{array} & \begin{array}{c} r_{1n}\\ r_{2n}\\ \vdots \\ 1 \end{array} \end{array}\right]=I+\tilde{R}\;,$$ (7)

where *I* is the auto-correlation matrix and $\tilde{R}$ is the co-correlation matrix, which represents the overlap of information in the multi-sensor system, $\lambda_{n}^{R}$, $\lambda_{n}^{I}$ and $\lambda_{n}^{\tilde{R}}$ denote the eigenvalues of *R*, *I* and $\tilde{R}$, respectively. It easy to see that, $\lambda_{n}^{R}$, $\lambda_{n}^{I}$ and $\lambda_{n}^{\tilde{R}}$ contain the information implied by *R*, *I* and $\tilde{R}$, respectively. Moreover, $\lambda_{n}^{R}$, $\lambda_{n}^{I}$ and $\lambda_{n}^{\tilde{R}}$ satisfy the following Eqs (8) and (9):

$$\lambda_{n}^{R}>0\;$$ (8)

$$\sum_{i=1}^{n}\lambda_{i}^{R}=\sum_{i=1}^{n}\lambda_{i}^{I}+\sum_{i=1}^{n}\lambda_{i}^{\tilde{R}}=n\;,$$ (9)

That is to say, the contribution of every sensor to the overall information of the multi-sensor system can be represented by the corresponding eigenvalues. From the information entropy perspective, the correlation information entropy of a multi-sensor system can be calculated as as given in Eq (10):

$$H_{R}=-\sum_{i=1}^{n}\frac{\lambda_{i}^{R}}{n}\log_{n}\frac{\lambda_{i}^{R}}{n}\;$$ (10)

Since the eigenvalues of the identity matrix *I* are 1, the overlap information entropy $H_{\tilde{R}}$ can be calculated as shown in Eq (11).

$$H_{\tilde{R}}=1-H_{R}=1+\sum_{i=1}^{n}\frac{\lambda_{i}^{R}}{n}\log_{n}\frac{\lambda_{i}^{R}}{n}=\sum_{i=1}^{n}\left(\frac{1}{n}+\frac{1+\lambda_{i}^{R}}{n}\log_{n}\frac{1+\lambda_{i}^{R}}{n}\right)\;$$ (11)

When $\lambda_{n}^{\tilde{R}}=0$ and $\lambda_{n}^{R}=\lambda_{n}^{I}=1$, the sensors provide completely different information, and the multi-sensor system information entropy is *H*_*R*_ = 1. There is no overlap of information. When $\lambda_{n}^{\tilde{R}}\neq 0$, the multi-sensor system does contain overlapping information, and $H_{\tilde{R}}>0$.

## Proposed method

In this paper, we use BPSO to find the feature subset with global dominance based on the overall evaluation of the combined effect of the features. During the optimization process, we study the optimization capabilities of different transfer functions as evaluation functions and choose the best transfer function. Because the focus of this paper is the evaluation of the combined effects of multiple features, the evaluation process considers the correlation between features and target classes as well as the redundancy of the feature set based on the correlation information entropy. The original feature space is represented as a multi-sensor system. Each feature is regarded as a sensor. In this environment, the correlation information entropy is used to judge the combined effect of each candidate subset and to reduce the redundant information among features. Based on this metric, an adaptive feature selection method based on V-shaped binary particle swarm optimization (VPFS) is proposed in this section. This method is different from traditional feature selection methods. For the first time, V-shaped binary particle swarm optimization is used to search the feature space, and the fitness function is constructed by using the correlation information entropy to evaluate each candidate subset. The algorithm finally obtains an adaptive-scale feature subset, without the need to set the size of the subset manually.

### Encoding

In this paper, each feature subset is encoded as a bit string. This encoding rule is widely applied in BPSO algorithms and other binary evolutionary algorithms. Given a feature set *F* with cardinality *n*, the length of the bit string is *n*. An individual is represented by an *n*-bit string. As shown in S1 Fig, if the ith bit is set to 1, then the corresponding feature is selected as part of the candidate feature subset; if the *i*th bit is set to 0, then the corresponding feature is not selected as part of the candidate feature subset.

With this coding rule, the feature selection problem is equivalent to finding the optimal bit string. The position of each particle is expressed as a discrete bit string, so the key component of the BPSO algorithm is the transfer function, which is used to obtain discrete positions from the real velocity values.

### Transfer functions for BPSO

A transfer function defines the probability of changing a position vector’s elements from 0 to 1 and vice versa. Transfer functions force particles to move in a binary space. According to Rashedi et al. [35], some concepts should be taken into account for selecting a transfer function in order to map velocity values to probability values as follows:

1. The range of a transfer function should be bounded in the interval [0,1], as they represent the probability that a particle should change its position.
2. A transfer function should provide a high probability of changing the position for a large absolute value of the velocity. Particles having large absolute values for their velocities are probably far from the best solution, so they should switch their positions in next iteration.
3. A transfer function should also present a small probability of changing the position for a small absolute value of the velocity.
4. The return value of a transfer function should increase as the velocity rises. Particles that are moving away from the best solution should have a higher probability of changing their position vectors in order to return their previous positions.
5. The return values of a transfer function should decrease as the velocity reduces.

Mirjalili S. and Lewis A. divided the transfer functions for BPSO into two classes: S-shaped functions and V-shaped functions [32]. The eight possible transfer functions are shown in detail in Table 1

**Table 1:** S-shaped and V-shaped families of transfer functions.

S-shaped function		V-shaped function	
Name	Transfer function	Name	Transfer function
S1	$\frac{1}{1+e^{-2v}}$	V1	$|\text{erf }(\frac{\sqrt{\pi }}{2}v)|$
S2	$\frac{1}{1+e^{-v}}$	V2	|tanh (*v*)|
S3	$\frac{1}{1+e^{\left(-v/2\right)}}$	V3	$|v/\sqrt{1+v^{2}}|$
S4	$\frac{1}{1+e^{\left(-v/3\right)}}$	V4	$|\frac{2}{\pi }arc\text{tan }(\frac{\pi }{2}v)|$

As seen in Table 1, S1, S2, S3 and S4 can be classified as S-shaped transfer functions, whereas V1, V2, V3, and V4 are V-shaped transfer functions. It can be seen that the function S2 is the sigmoid function used in traditional BPSO. This transfer function is the most widely used function for BPSO. The curves of S-shaped transfer functions are shown in S2 Fig, while the curves of V-shaped transfer functions are shown in S3 Fig. As shown in S2 Fig, S1 returns the highest probability among them for the same value of velocity.The V-shaped transfer functions shown in S3 Fig are different from the S-shaped transfer functions, which do not force particles to take a value of 0 or 1. The advantage of a V-shaped transfer functions is that they encourage particles to remain in their current positions when their velocity values are low or to switch to their complements when their velocity values are high.

Mirjalili S. has proven the optimization effects of V-shaped and S-shaped transform functions on benchmark functions in optimization tests and has verified the superior search capability of V-shaped transform functions on many benchmark functions. For the fitness function proposed in this paper, we test for the optimal function among these eight functions for feature selection and choose the best transfer function in our experimental study. The results are discussed in detail in section 4.

### Fitness function

In this paper, correlation information entropy theory is used to evaluate the combined effect of each candidate feature subset. Each particle in the swarm is regarded as a candidate feature subset, and each bit in a particle string is regarded as one feature dimension. The original feature set *F* = {*f*_1_,*f*_2_, …,*f*_*n*_} is a multivariable system with *n* variables, where each feature *f*_*i*_ corresponds to an equivalent sensor, as described above. Unlike in the original model of a multi-sensor system, this paper does not use specific data on instances of *n*-dimensional features as the input; instead, instances of the target classes *C* = {*c*_1_,*c*_2_, …,*c*_*m*_} are taken as the system time series. Based on the correlation between the features and target classes, a multivariable model *M* is constructed, where *M* is equivalent to the matrix *R* in section 1.3. The feature information system *M* is formed as in Eq (12):

$$M=\left[\begin{array}{cccc} \begin{array}{c} I_{11}\\ I_{21}\\ \vdots \\ I_{n1} \end{array} & \begin{array}{c} I_{12}\\ I_{22}\\ \vdots \\ I_{n2} \end{array} & \begin{array}{c} \ldots \\ \ldots \\ \ldots \end{array} & \begin{array}{c} I_{1m}\\ I_{2m}\\ \vdots \\ I_{\text{nm}} \end{array} \end{array}\right]\;$$ (12)

Of particular interest is the element Iij, which represents the mutual information between the *i*th feature and the *j*th target class; this marks the most significant difference from a multi-sensor system. Since the correlation information entropy in a multi-sensor system is focused on the redundancy (overlap) relationship among the variables, it is impossible to consider the correlation between features and target classes if data on instances of each feature are taken as the input. The utilization of mutual information as the elements in *M* enables the consideration of not only feature redundancy but also the correlation between features and target classes. Moreover, this model also can reduce the size of the multivariable system and thus satisfies the basic requirements of feature selection. The mutual information is calculated as given in Eqs (13) and (14):

$$I\left(f_{i};\;c_{j}\right)=H\;\left(f_{i}\right)+H\;\left(c_{j}\right)-H\;\left(f_{i},c_{j}\right)\;$$ (13)

$$H\;\left(f_{i},c_{j}\right)=-\sum_{f_{i}}p(f_{i},c_{j})lbp\left(f_{i},c_{j}\right)\;$$ (14)

The information entropy of the variable *v* is calculated using Eq (15):

$$H\left(v\right)=-\sum_{i=1}^{V}p\left(v_{i}\right)\text{ lb}p\left(v_{i}\right)\;$$ (15)

Without loss of generality, the normalization and centralization of *M* are performed as shown in Eqs (16) and (17) to obtain the matrix $Q_{F}=\left[{\bar{I}}_{ij}\right]$.

$${\hat{I}}_{ij}=I_{ij}-\frac{\max\;I_{i}-\min\;I_{i}}{2}\;$$ (16)

$${\bar{I}}_{ij}=\frac{{\hat{I}}_{ij}}{\sqrt{\sum_{j=1}^{m}{\left({\hat{I}}_{ij}\right)}^{2}}}\;$$ (17)

The correlation matrix is calculated as in Eq (18):

$$Rel={Q_{F}}^{T}\cdot Q_{F}\;$$ (18)

The correlation matrix *Rel* exhibits symmetry, and each element in the matrix represents the similarity of the information provided by the two corresponding features with regard to the target classes. This process enables the initial compression of the original data space of *k* × *n* into a matrix of *m* × *n* (*m* ≪ *n*, *n* ≪ *k*). Although the matrix dimensions increase to *n* × *n* when the correlation matrix is calculated, the data space is nevertheless greatly reduced.

The eigenvalue $\lambda_{n}^{Rel}$ of the correlation matrix *Rel* is calculated to obtain the correlation information entropy *H*_*Rel*_ according to Eq (11).The smaller the degree of feature redundancy $H_{\tilde{Rel}}$ is, the higher the degree of independence among the features is, and the greater is the relevance between the candidate feature subset and the target classes. Similarly, when $H_{\tilde{Rel}}=0$ and *H*_*Rel*_ = 1, there is no redundant information in the system, and every feature provides different information. When $H_{\tilde{Rel}}>0$ and *H*_*Rel*_ < 1, the degree of feature redundancy can be represented by the value of $H_{\tilde{Rel}}$. Thus, the mapping from the multi-sensor system to the feature selection space is complete. Because of the calculation of eigenvalues, we need to delete features with the same value in all classes before calculating the correlation matrix to avoid anomalies. For the purpose of feature selection, a feature that has the same value in all classes provides no guidance for the learning task and is, in essence, a useless feature.

An analysis of why the metrics discussed above can consider both correlation and redundancy is presented as follows. From the perspective of linear space, the decomposition of an *n*-order symmetric square matrix produces *n* standard orthogonal bases of a linear space defined by inner products. Then, the matrix is projected onto these *n* standard orthogonal bases. The *n* eigenvectors correspond to the *n* standard orthogonal bases, and the size of each eigenvalue represents the length of the projection of the matrix onto the corresponding basis. The larger an eigenvalue is, the larger is the variance of the matrix in the corresponding eigenvector, and the greater is the power of that basis. In the task of data mining, the largest eigenvalue corresponds to the feature vector that contains the largest amount of information. In this paper, the correlation information entropy is used to evaluate the combined effect of the features in each candidate feature subset. The eigenvalues, which are based on the mutual information of each candidate subset, measure the correlation. The redundancy among features is also measured, by $H_{\tilde{Rel}}$.

In the greedy algorithm to be verified, if *H*_*Rel*_ is close to 1, then only very few features can be used if non-redundancy is to be ensured. However, because of the advantages of VBPSO, we can find the combination of different features that most closely approximates 1. Therefore, two new fitness functions are constructed based on the above theory in the following Eqs (19) and (20):

$$Fitness1\;\left(h\right)=\left|\left(1-H_{Rel\left({}_{h}\right)}\right)-\delta \right|\;$$ (19)

$$Fitness2\;\left(h\right)=e^{\left|\left(1-H_{Rel\left({}_{h}\right)}\right)-\delta \right|}\;$$ (20)

where *h* denotes the *h*th particle in the particle swarm and *H*_*Rel*(*h*)_ denotes the correlation information entropy of the candidate subset represented by particle *h*. *Rel* is a matrix of size *n* × *n*. If *n* is 10 and particle *h* is 0101001011, then the fitness is calculated using a correlation matrix *Rel*(*h*) of size 5 × 5, corresponding to the five features dimensions 2, 4, 7, 9, and 10. The significance of *δ* is that it is the information control parameter, which is used to control the maximum amount of redundant information that a candidate subset can contain. When *δ* = 0, the fitness functions will attempt to keep the redundancy of the desired subset close to zero.

When the dimensions are few, the particle optimization process is relatively stable, so Eq (19) is suitable for avoiding immature convergence on datasets of small-and medium-scale dimensions, whereas Eq (20) is suitable for high-dimensional and ultra-high-dimensional datasets. The significance of the exponent *e* is that a curve with a higher exponent is more magnified in contrast to a linear equation, and the amplification of the difference provides better guidance for long-bit particles.

### VPFS

The VPFS algorithm uses the correlation information entropy for subset evaluation and the heuristic evolutionary search algorithm known as VBPSO as the search strategy. The VPFS algorithm, compared with traditional feature selection methods, has the following advantages: 1) The correlation and redundancy among all features in each subset are evaluated simultaneously rather than for pairs of features. 2) This algorithm represents the first use of V-shaped binary particle swarm optimization as the search strategy in a feature space. These two innovations enable improvements to the subset evaluation function and search strategy used during feature selection. The superior ability of the transfer function V4 and the optimal specific value of *δ* will be demonstrated in detail in the experimental section. General diagram about the overall algorithm is included in S4 Fig. The specific process of the algorithm is shown in Algorithm 1:

**Algorithm 1**. VPFS algorithm.

**Input**: Data set *D*, feature set *F*, target classes *C*

**Output**: Adaptive feature set *S*

1) **For** each *f*_*i*_ ∈ *F*, *c*_*j*_ ∈ *C*:

2)  Calculate *I*_*ij*_ to get the matrix *M*;

3) **End For**

4) Obtain the matrix *Q*_*F*_ by the centralization and standardization;

5) *Rel* = *Q*_*F*_^*T*^ · *Q*_*F*_;

6) **Initialization**: particles

7) **While** (*maximum iterations is not attained*)

8)  **For** each particle

9)   Calculate the fitness according to *H*_*Rel*(*h*)_;

10)   Update *pbest* and *gbest*;

11)   Update $v_{h}^{k}(t+1)$ and $x_{h}^{k}(t+1)$;

12)  **End For**

13) **End While**

14) S ← *gbest*.

## Experimental results and analysis

To verify the effectiveness of the proposed method, an experiment is performed on UCI’s machine learning data repository. Firstly, the eight different transfer functions are tested for BPSO. Based on the optimization results for the proposed fitness functions, the best transfer function is applied for subsequent feature selection. The purpose of the second part of the experiment is to compare the VPFS method with other methods on various classifiers. The classification performance and differentiation are described in detail based on five indicators. Finally, the execution times are presented to illustrate the time efficiency on different datasets. The experimental workbench is MATLAB 2013. For the continuous data, we use the MDL data discretization method implemented in Weka 3.8. The details of datasets are shown in Table 2.

**Table 2:** Descriptions of UCI benchmark datasets.

No.	Dataset	Number of Instances	Number of Features	Number of classes	Scientific area
1	Breast Cancer	569	32	2	Biology
2	Dermatology	366	33	6	Biology
3	Soybean	683	35	19	Biology
4	QSAR	1055	41	2	Chemometrics
5	Synthetic Control	600	60	6	Computer
6	Mice Protein	1080	82	8	Biology
7	Gas Sensor Array	13910	129	6	Computer
8	Musk	6598	168	2	Physical
9	Multi-feature pixel	2000	240	10	Computer
10	Isolet	1559	618	26	Computer

Breast Cancer is a cancer diagnostic dataset. It consists of 569 instances, which are uniformly distributed between 2 categories, and 32 feature dimensions. Dermatology is a dermatological data set with 33 feature dimensions and 366 instances that are heterogeneously distributed among 6 categories. Soybean is a soybean disease diagnostic dataset, with 19 categories and an uneven distribution. There are only 26 instances in the smallest category and 149 instances in the largest category. QSAR is a biodegradation data set constructed in 2013 with no missing values, which contains 1/3 positive instances and 2/3 negative instances. Synthetic Control is a control chart data set in which 600 instances with 60 feature dimensions are distributed evenly among 6 categories. Mice Protein is a mouse protein expression data set published in 2015, in which each instance has 82 feature dimensions. Gas Sensor Array is a gas sensor array drift data set from 2013 with no missing values, in which 13910 instances are evenly distributed among 6 categories. Musk is a data set with no missing data in which the negative instances are approximately 5 times greater in number than the positive instances. Multi-feature Pixel is a part of the data set consisting of handwritten instances of the numerals “0” to “9” in Multi-feature, which possesses up to 240 feature dimensions. Isolet is drawn from a dataset consisting of 150 testers’ pronunciations of the 26 English letters. For this paper, the Isolet5 collection is selected, which has an enormous number of feature dimensions but contains only 1559 instances. These datasets contain various numbers of features, from 32 to 618, and various numbers of target classes, from 2 to 26. The 10 data sets listed above are taken from the fields of medicine, biology, image processing, audio processing and industry, among others.

### Comparison of transfer functions

To ensure a fair assessment, we select six data sets from Table 2 on which to test the eight functions using Eq (19). The same fixed initialization parameters are specified, as follows: 500 generations and *c*_1_ = *c*_2_ = *w* = 2. The final optimal search results (minimum values) obtained by taking the optimal average of 30 rounds for each of the different transfer functions are shown in Table 3, where the first column indicates the data set as presented in Table 2 and the subsequent columns correspond to the different transfer functions. The data presented in the table are the mean values. The number presented in bold normal font represents the optimal minimum value among the optimization results for the eight functions. The number presented in bold italic font is the next-to-optimal minimum value among the optimization results for the eight functions.

**Table 3:** Minimization results for fitness function 1 using different transfer functions.

Dataset	Transfer function							
	S1	S2	S3	S4	V1	V2	V3	V4
Dermatology	5.85E-06	5.11E-06	9.83E-06	9.27E-06	5.39E-06	4.61E-06	***4.37E-06***	**3.61E-06**
Soybean	6.59E-03	1.36E-02	5.60E-02	9.47E-02	***1.86E-03***	2.58E-03	1.94E-03	**9.87E-04**
Synthetic control	3.47E-04	3.75E-03	4.39E-02	8.20E-02	4.29E-05	***4.10E-05***	4.26E-05	**3.74E-05**
Mice Protein	9.57E-05	6.91E-04	1.52E-02	3.10E-02	***7.42E-06***	8.90E-06	1.01E-05	**5.74E-06**
Pixel	5.88E-02	7.39E-02	7.64E-02	8.08E-02	5.44E-06	7.77E-06	***4.69E-06***	**4.53E-06**
Isolet	8.86E-02	9.43E-02	9.36E-02	9.27E-02	4.95E-06	***4.55E-06***	5.39E-06	**2.71E-06**

According to Eq (19), when the value of the function is closer to 0, the redundant information provided by the feature subset is closer to *δ*. Therefore, the smaller *δ* is, the less redundant information there is. To prove that the function optimization process is not affected by *δ*, the value of *δ* used in the function is randomly set to a value of 0 or [0,0.5]. The results show that the V-shaped transfer functions are superior to any S-shaped transfer function, with the exception that the optimal value of S2 is smaller than that of V1 on the Dermatology data set. In particular, the transfer function V4 achieves the best average fitness value on all data sets, showing a strong ability to find the optimal results, whereas the next-to-optimal solutions are found using V3, V1, V2, V1, V3 and V2. In other words, the other three V-shaped transfer functions perform similarly to but more weakly than V4. Among the S-shaped transfer functions, the function S1 performs better than the others.

To more intuitively illustrate the excellent performance of V4, S5 and S6 Figs present the convergence curves within a single round for each of the eight functions on the Synthetic Control and Mice Protein datasets separately. Unlike the data in Table 3, in this validation, the fitness function is altered to Eq (20) to graphically illustrate the optimization capability of the V4 function for feature subsets. The results for 500 iterations executed with the same initialization parameters are presented. For this analysis, the value of *δ* is 0.

We can see that according to the fitness function given in Eq (20), the fitness value approaches 1 when the redundant information in a feature subset approaches zero. The graphical results show that the transfer function V4 still exhibits the best search ability. The function S1 is superior to the other S-shaped transfer functions, and S3 is the worst. The graph yields the same conclusion as Table 3. Therefore, in subsequent experiments, V4 is used as the transfer function for mapping the positions of the particles.

The function V4 is excellently well suited for use with the fitness function proposed in this paper. However, the S-shaped functions, especially S2, are generally superior to the V-shaped functions on the Realization Instances set for the combined auction problem. Therefore, for the selection of the transfer function, the performances of the different functions should be tested for the specific problem of interest.

Since the proposed method depends on some parameters specified in experiments, an analysis regarding the robustness to parameter specifications is required. There are two important parameters influence results of the proposed method, which are the probability *p* of initializing to 1 bit and inertia weight *w*. Probability p affects the size of the adaptive subset, The inertia weight affects the best fitness value. Specific changes can be shown in Table 4. In this testing, we fixed the other values of variables and adjusted values of only one variable on four datasets. The observation of results is got by taking the average value in 30 runs. With the increase of the probability, the size of the feature subset is expanding. So the robustness to the probability is not ideal enough. However, when the inertia weight is 1.5 or 2, the best fitness value is the minimum. Since the gap of fitness values are quite small by taking 1.5 and 2 respectively, the inertia weight is robust.

**Table 4:** Parameter testing of probability *p* and inertia weight *w*.

*p*	Number of Selected Features				*w*	Fitness Value			
	Dermatology	QSAR	Synthetic	Pixel		Dermatology	QSAR	Synthetic	Pixel
0.1	3	4	5	19	0.5	6.09E-06	1.17E-02	1.04E-02	8.62E-06
0.2	4	10	8	36	1.0	4.52E-06	9.15E-03	9.97E-03	4.69E-06
0.3	8	14	13	58	1.5	4.39E-06	**7.85E-06**	**2.51E-05**	2.94E-06
0.4	10	16	20	89	2.0	**3.61E-06**	1.76E-05	3.74E-05	**2.27E-07**
0.5	13	20	27	96	2.5	4.54E-06	1.26E-04	8.33E-05	3.05E-07

### The classification performance of VPFS

This section verifies the performance of the feature subsets derived using the VPFS method in terms of specific classifiers. Six different methods are considered for comparison: FCBF, IG, ReliefF, mRMR, SFS and CMFS-*η*. FCBF and CMFS-*η* are adaptive subset selection methods. IG, ReliefF, mRMR are ranking methods. SFS is inherently an adaptive subset selection method, but in this paper, it is transformed into a ranking method by adjusting the neighborhood and dependency. To validate the generalization capability of the proposed method, SVM, 1-NN and Naïve Bayes classifiers are used to study the selected subsets. Classification is performed by means of 10-fold cross validation. The experimental datasets are the 10 high-dimensional datasets introduced in Table 2.To make the comparison more insightful, the different methods should be compared with the case that all features are used in classification process when SVM, 1-NN, and Naïve Bayes Classifiers are employed respectively. This comparison will illustrate the pros and cons of feature selection methods. Therefore, we first give the classification accuracy of ten datasets on all features in Table 5.

**Table 5:** Comparison of classification accuracy for three classifiers on full set.

No.	Classification Accuracy/%		
	SVM	1-NN	Naïve Bayes
Breast Cancer	97.92	95.96	92.97
Dematology	95.35	94.54	97.54
Soybean	93.85	91.22	92.97
QSAR	85.59	84.46	75.92
Synthetic Control	99.17	96.50	94.67
Mice Protein	100	99.26	87.50
Gas Sensor Array	97.14	99.47	59.47
Musk	94.92	95.80	83.86
Multi-feature Pixel	97.55	96.15	93.3
Isolet	96.81	89.58	84.21
Avg A	*95.83*	*94.29*	*86.24*

The experimental results regarding classification accuracy of different feature selection methods are shown in Tables 6–8. To ensure a fair comparison, the parameters of the algorithm are set such that it yields a subset of the same size as that selected by the CMFS-*η* algorithm. The value of *η* is 0.35 in the CMFS-*η*. Since the proposed algorithm is evolutionarily optimized, the data labeled as VPFS-Avg in the table represent the average classification accuracy rate and average selected subset size for 20 runs, whereas VPFS-Best represents the best classification accuracy rate and the corresponding subset size among the 20 runs. The data presented in Tables 6–8 show the classification accuracy rate for the subset selected by each feature selection method for the corresponding data set using each type of classifier, and the bold values represent the highest accuracy rates in their respective rows. The data shown in parentheses indicate the size of the selected subset. For the Dermatology and Soybean datasets, we use Eq (19) as the fitness function, whereas Eq (20) is used for the other datasets. The values of *δ* used in VPFS for the 10 data sets, in the order in which they are listed in the tables, are 0.1,0,0,0,0.25,0,0,0,0,0, respectively. It can be seen that the evolutionary algorithm is almost independent of *δ*, with the exception that non-zero *δ* values are specified for two data sets to obtain subsets of approximately the same size as those found by the other methods. For most data sets, however, this value can be set directly to *δ* = 0, which is very different from the control required for the greedy algorithm in CMFS-*η*. The probabilities of initializing to 1 bit are 0.3, 0.55, 0.55, 0.55, 0.3, 0.3, 0.15, 0.1, 0.1, 0.1, respectively. Here, IG, ReliefF, mRMR and SFS select sorted subsets of equal size.

**Table 6:** Comparison of classification accuracy for the SVM classifier.

No.	Classification Accuracy/%(Number of Features in Subset)Rank of Classification Accuracy							
	FCBF	IG	ReliefF	mRMR	SFS	CMFS-*η*	VPFS-Avg	VPFS-Best
1	95.78(2.5)	93.50(6)	93.32(7)	94.55(5)	95.78(2.5)	94.90(4)	96.17(1)	**97.19**
2	95.67(3)	85.25(7)	95.36(4)	93.99(5.5)	93.99(5.5)	97.00(2)	97.87(1)	**98.36**
3	91.80(5)	92.83(2)	92.68(3)	92.97(1)	90.19(6)	90.04(7)	92.31(4)	**93.7**
4	73.74(7)	83.32(4)	82.46(5)	80.47(6)	84.08(2)	83.98(3)	84.99(1)	**85.78**
5	82.67(4)	72.33(7)	77.00(5)	73.83(6)	93.83(2)	90.83(3)	94.86(1)	**97.50**
6	96.29(3)	99.73(2)	**99.81**(1)	95.28(4)	93.25(6)	92.50(7)	94.45(5)	96.48
7	84.06(7)	84.14(6)	84.28(5)	95.14(2)	85.44(4)	93.67(3)	97.86(1)	**99.47**
8	84.58(7)	91.06(4)	88.31(5)	87.59(6)	93.48(2)	92.54(3)	94.59(1)	**95.33**
9	93.90(5)	90.45(7)	92.10(6)	94.40(4)	94.95(2)	94.90(3)	96.22(1)	**97.25**
10	84.67(3)	73.32(6)	51.21(7)	85.54(1)	80.56(5)	82.48(4)	85.12(2)	**88.14**
***Avg A***	*88.32*	*86.59*	*85.65*	*89.38*	*90.56*	*91.28*	*93.44*	***94.92***
***WTL***	9/0/1	8/0/2	8/0/2	7/0/3	10/0/0	10/0/0		
***Avg R***	4.65	5.1	4.8	4.05	3.7	3.9	1.8	
***F-test***	*χ*^2^ = 15.46	*p* = 0.017						
***Post***(***p***)	0.0032	0.0006	0.0019	0.0198	0.0486	0.0295		

**Table 7:** Comparison of classification accuracy for the 1-NN classifier.

No.	Classification Accuracy/%(Number of Features in Subset)Rank of Classification Accuracy							
	FCBF	IG	ReliefF	mRMR	SFS	CMFS-*η*	VPFS-Avg	VPFS-Best
1	94.55(4.5)	94.55(4.5)	94.20(6)	93.32(7)	94.38(3)	94.90(2)	95.06(1)	**95.96**
2	95.36(1)	83.33(7)	94.26(2)	93.44(4)	89.87(5)	88.25(6)	93.96(3)	**95.63**
3	86.38(5)	86.53(4)	89.02(3)	83.31(7)	83.89(6)	89.31(2)	89.59(1)	**92.83**
4	81.13(7)	82.08(5)	83.13(2)	82.94(3.5)	82.94(3.5)	81.52(6)	83.14(1)	**84.83**
5	80.50(7)	83.00(6)	84.00(4)	85.67(3)	83.17(5)	89.33(2)	91.13(11	**93.17**
6	94.63(6)	**99.9** (1.5)	**99.9**(1.5)	98.52(3)	70.37(7)	97.50(5)	98.00 (4)	99.26
7	99.22(3)	**99.63**(1)	99.30(2)	97.64(6)	98.92(4)	95.69(7)	98.48(5)	99.31
8	93.14(7)	**95.63**(1)	93.66(5)	94.14(4)	93.45(6)	94.60(2)	94.40(3)	94.68
9	92.80(2)	84.4(7)	86.75(6)	90.40(5)	92.25(4)	93.75(1)	92.60(3)	**94.8**
10	66.45(6)	68.30(4)	48.93(7)	80.07(1)	71.07(3)	67.12(5)	74.88(2)	**79.17**
***Avg A***	*88.42*	*87.74*	*87.32*	*89.95*	*86.03*	*89.2*	*91.12*	***92.96***
***WTL***	7/0/3	7/0/3	7/0/3	8/0/2	9/0/1	9/0/1		
***Avg R***	4.85	4.1	3.85	4.35	4.65	3.8	2.4	
***F-test***	*χ*^2^ = 8.36	*p* = 0.2131						
***Post***(***p***)	0.0112	0.0769	0.1289	0.0431	0.0198	0.1418		

**Table 8:** Comparison of classification accuracy for the naïve bayes classifier.

No.	Classification Accuracy/%(Number of Features in Subset)Rank of Classification Accuracy							
	FCBF	IG	ReliefF	mRMR	SFS	CMFS-*η*	VPFS-Avg	VPFS-Best
1	95.08(2)	94.73(3)	92.79(7)	94.55(4)	94.38(5.5)	94.38(5.5)	95.52(1)	**96.66**
2	96.98(1)	86.89(7)	96.72(3)	96.45(4.5)	94.81(6)	96.45(4.5)	96.83(2)	**98.09**
3	90.04(2)	87.99(5)	89.31(3.5)	89.31(3.5)	85.94(7)	86.68(6)	91.21(1)	**92.83**
4	63.98(7)	73.65(6)	75.17(5)	75.83(4)	77.44(3)	81.61(2)	81.73(1)	**82.46**
5	80.00(6)	77.67(7)	80.67(5)	82.67(4)	94.50(1)	91.12(3)	94.29(2)	**96.83**
6	94.07(3)	**98.52**(1)	98.42(2)	83.89(4)	75.46(7)	82.68(6)	83.42(5)	87.96
7	55.75(7)	65.86(4)	61.92(6)	77.89(2)	62.06(5)	68.03(3)	82.92(1)	**85.75**
8	76.46(7)	86.47(4)	84.72(5)	84.59(6)	89.13(3)	90.65(2)	90.95(1)	**91.92**
9	91.15(2)	82.7(7)	84.25(6)	88.15(5)	91.40(1)	90.45(4)	90.99(3)	**92.55**
10	84.22(2)	55.23(6)	35.23(7)	68.77(5)	70.37(4)	82.80(3)	85.17(1)	**89.10**
***Avg A***	*82.77*	*80.97*	*79.92*	*84.21*	*83.55*	*86.49*	*89.3*	***91.42***
***WTL***	7/0/3	9/0/1	9/0/1	9/0/1	8/0/2	10/0/0		
***Avg R***	3.9	5	4.95	4.2	4.25	3.9	1.8	
***F-test***	*χ*^2^ = 14.71	*p* = 0.0226						
***Post***(***p***)	0.0295	0.0009	0.0011	0.0129	0.0112	0.0295		

In this paper, we use several statistical indicators [36] to evaluate the different feature selection algorithms. The *Avg* *A* rows of the tables represent the average classification accuracy of each algorithm on the 10 data sets. The *WTL* row represents the number of wins / ties / losses for VPFS-Avg in comparison with the method corresponding to the indicated column on the 10 data sets. The overall winning percentage is calculated as in Eq (21):

Pwin=∑WVPFS∑WVPFS∑WTL′∑WTL′ (21)

*WTL*’ represents the overall number of VPFS-Avg evaluations compared with the other six methods on the 10 data sets (60 in this paper), and *W*_*VPFS*_ represents the total number of wins. The value after the brackets in Tables 6–8 is the rank in terms of classification accuracy on one of the 10 data sets. The *Avg* *R* values in tables indicate the average rank in terms of classification accuracy achieved by the different feature selection methods on the 10 data sets.

The Friedman test is a nonparametric test for the existence of significant differences between population distributions, which is abbreviated as *F-test* in Tables 4–6. The global differences among the seven different methods are evaluated in the tables. The larger the value of *χ*^2^ is, the greater is the global difference among the methods. When *p* is less than 0.05, the difference is considered significant. The specific calculation is as shown in Eq (22):

$$\chi_{{}^{F}}^{2}=\frac{12N}{k\;\left(k+1\right)}\left[\sum_{j}R_{j}^{2}-\frac{k{\left(k+1\right)}^{2}}{4}\right]\;$$ (22)

where *R*_*i*_ is the *Avg R* of the *i*th method, *N* is the number of data sets (*N* = 10), and *k* is the number of feature selection algorithms (*k* = 7). The value of *p* can be obtained from a *χ*^2^ look-up table.

In addition to the global difference, *Post-hoc* (*p*) values are used to compare the differences between pairs of methods, which is abbreviated as *Post*(*p*) in Tables 6–8. The corresponding row shows the differences between VPFS-Avg and the other six methods. When the value of *p* is less than 0.05, the difference between the two feature selection methods is considered significant. The specific calculation is as give in Eq (23):

z=Rj-RVPFSRj-RVPFSkk+16Nkk+16N (23)

where *R*_*VPFS*_ is the *Avg R* of the VPFS-Avg results and *N*, *k* and *R*_*i*_ have the same meanings as above. *Post-hoc* (*p*) values can be obtained by querying the standard normal distribution table for *z* values.

Table 6 shows the performance of each feature selection method when the SVM classifier is used. It can be seen that VPFS-Best achieves the highest classification accuracy on nine of the data sets and the highest average classification accuracy. VPFS-Avg achieves the next-to-optimal classification accuracy on 8 data sets and the next-to-optimal average classification accuracy. On the Soybean data set, although VPFS-Avg does not achieve the next-to-optimal classification accuracy, it is only 0.52% behind the next-to-optimal value achieved by IG. The performance quality of VPFS on the Mice Protein dataset is only moderate. On this dataset, ranking methods perform better than adaptive subset selection methods. The advantages of the proposed method are more prominent for the ultra-high-dimensional Multi-feature Pixel and Isolet data sets. VPFS-Avg achieves the highest overall average ranking of 1.8. For the SVM classifier, the global difference *p* is 0.0111, which indicates that the seven methods show significant differences. In the comparisons of VPFS with FCFS, IG, ReliefF, mRMR, SFS and CMFS-*η*, the *Post-hoc* (*p*) values are all smaller than 0.05. This shows that the proposed method performs significantly differently from each of other methods when the SVM classifier is used.

As seen from the results for the 1-NN classifier presented in Table 7, VPFS-Best achieves the highest classification accuracy on seven of the data sets and the highest average classification accuracy. VPFS-Avg achieves the next-to-optimal classification accuracy on only four data sets and the next-to-optimal average classification accuracy. However, VPFS-Avg still achieves the highest *Avg R*. On the Multi-feature Pixel dataset, it is only 1.25% behind the sub-optimal SFS algorithm. For the 1-NN classifier, the global difference *p* is 0.2131, which indicates that the seven methods show no significant difference when this classifier is used; all seven yield feature subsets with similar effects on the classification results. The values of *Post-hoc* (*p*) are all smaller than 0.05 for the comparisons of VPFS with FCBF, IG, mRMR and SFS. However, as indicated by *Post-hoc* (*p*) values of 0.1418 and 0.1289, the proposed method performs less differently from CMFS-*η* and ReliefF when the 1-NN classifier is used.

Upon comparing the experimental results for the Naïve Bayes classifier presented in Table 8, we can again see the advantages of VPFS in terms of classification performance. VPFS-Best achieves the best performance on nine of the datasets. The average classification accuracy of VPFS-Avg is almost 5% higher than that of CMFS-*η*, which achieves the next-to-optimal average classification accuracy. VPFS-Avg again achieves the highest overall average ranking of 1.8. The performance of the proposed method in combination with the Naïve Bayes classifier is similar to that in combination with the SVM classifier in terms of *χ*^2^. For the Naïve Bayes classifier, the global difference *p* is 0.0078, indicating that the seven methods show significant differences. Moreover, the VPFS method performs significantly differently from all other methods, as indicated by the fact that the values of *Post-hoc* (*p*) are all smaller than 0.05.

The above experimental analysis and the data presented in Tables 6–8 show that VPFS-Best achieves the best effect in combination with all three classifiers, whereas VPFS-Avg achieves the overall next-to-optimal results. Comparisons with FCBF, IG, ReliefF, mRMR, SFS and CMFS-*η* in terms of the WTL results reveal that the overall winning percentages of the proposed method are 86.67%, 78.33% and 86.67% for the SVM, 1-NN and Naïve Bayes classifiers, respectively. In the classification task, VPFS and CMFS-*η*, which use the correlation information entropy measure, are superior to FCBF, IG, ReliefF, mRMR and SFS. As seen from the fact that it enables the best classification accuracies, VPFS can find superior feature combinations compared with those identified by the other methods. Thus, the proposed method is more suitable for classification tasks. VPFS-Avg represents the average performance on each classifier. VPFS-Best represents the best performance on each classifier. In addition to the above four indicators, another important indicator is the compression ratio, which can indicate the trade-off of feature selection algorithms in terms of prediction accuracy and the compactness. The compression ratio is calculated as in Eq (24):

$$Compression\;Ratio=\left(1-\frac{\left|Subset\right|}{\left|Full\;Set\right|}\right)\%$$ (24)

where |*Subset*| is the cardinality of selected features and |*Full Set*| is the cardinality of whole dataset. Because the proposed method works in the filter approach, we employed SVM, 1-NN, and Naïve Bayes Classifiers to verify the prediction accuracy based on selected feature subsets. It is necessary to examine the performance regarding the ratio of features selected over all features in Table 9. Tha *Avg C R* row represents the average compression ratio of corresponding method in ten datasets. FCBF method has achieved the highest compression ratio and CMFS method is the suboptimal performer. Other five methods are very similar to the compression ratio. According to the results of classification accuracy in Tables 6–8, there are competitive advantages of FCBF and VPFS, which are both subset selection methods. In ranking methods as mentioned above, CMFS–*η* is best performer. In general terms, the performance of subset selection methods is better than ranking methods on classification accuracy and compression ratio.

**Table 9:** Comparison of compression ratio.

No.	Number of Features in Subset(Compression Ratio/%)							
	FCBF	IG	ReliefF	mRmR	SFS	CMFS-*η*	VPFS-Avg	VPFS-Best
1	7(**78.13**)	7(**78.13**)	7(**78.13**)	7(**78.13**)	7(**78.13**)	7(**78.13**)	7(**78.13**)	7(**78.13**)
2	16(51.52)	16(51.52)	16(51.52)	16(51.52)	16(51.52)	13(**60.61**)	16(51.52)	16(51.52)
3	16(54.29)	16(54.29)	16(54.29)	16(54.29)	16(54.29)	12(**65.71**)	17(51.43)	16(54.29)
4	5(**87.80**)	23(43.90)	23(43.90)	23(43.90)	23(43.90)	23(43.90)	22(46.34)	21(48.78)
5	15(75.00)	14(76.67)	14(76.67)	14(76.67)	14(76.67)	11(**81.67**)	12(80.00)	14(76.67)
6	17(**79.27**)	36(56.10)	36(56.10)	36(56.10)	36(56.10)	28(65.85)	35(57.32)	36(56.10)
7	12(**90.70**)	14(89.15)	14(89.15)	14(89.15)	14(89.15)	14(89.15)	13(89.92)	13(89.92)
8	6(**96.43**)	12(92.86)	12(92.86)	14(91.67)	14(91.67)	12(92.86)	14(91.67)	15(91.07)
9	27(**88.75**)	58(75.83)	58(75.83)	58(75.83)	58(75.83)	44(81.67)	59(75.42)	60(75.00)
10	31(**94.98**)	49(92.07)	49(92.07)	49(92.07)	49(92.07)	39(93.69)	49(92.07)	55(91.10)
***Avg C R***	***89.43***	*82.96*	*82.96*	*82.82*	*82.82*	*85.88*	*83.03*	*82.41*

We can summarize the proposed method in terms of the following two aspects: the search strategy and the evaluation function. Considering that the correlation information entropy is used in both VPFS and our previously proposed method, namely, CMFS-*η*, the superior performance of VPFS compared with that of CMFS-*η* shows that the V-shaped binary particle swarm optimization approach is better than the simple greedy search strategy. Since VPFS and FCBF are both subset selection methods, the superior performance of VPFS and CMFS-*η* compared with that of FCBF shows that evaluation based on the correlation information entropy is better than evaluation based on the basic information entropy. With regard to overall stability, the VPFS method is the most stable in overall performance for each classifier and on each dataset. However, because of the limitations related to the generation and initialization processes in the evolutionary search algorithm, the feature subsets obtained by VPFS may be only approximations of the global optimal solutions. Moreover, when there are multiple global optimal solutions, the proposed method will not always yield the same selected subset.

### Run-time performance comparison

The efficiency of the calculation is also an important indicator when evaluating different feature selection methods. The purpose of feature selection is to reduce the dimensionality of the original data and to improve the efficiency of subsequent learning tasks. However, if the feature selection method also requires considerable time consumption, then such an effort will be meaningless. CoFS is a feature selection method based on a cooperative game that was proposed in [10]. This method considers the combined effect of all features in a subset and achieves excellent classification performance. However, this method has a high time complexity for the generation of candidate features. The time complexity of this algorithm is exponential, which leads to high time consumption. The mRMR method is of similar time complexity to the CMFS-*η* algorithm proposed in our previous work. The run time of SFS depends on its neighborhood and dependency; when the neighborhood and dependency are very small, the algorithm becomes relatively time-consuming. The run times of the various methods are shown in Table 10.

**Table 10:** Run times on UCI datasets.

Dataset	Running Time/s				
	CoFS	mRMR	CMFS-*η*	SFS	VPFS
Synthetic Control	1.06	0.31	0.16	0.27	1.01
Multi-Feature Pixel	44.37	22.28	2.48	15.09	15.92
Isolet	124.02	57.95	42.17	90.92	18.86

In the same environment, consider the results achieved by five methods on three data sets of increasingly high dimensionality. The parameters of the VPFS method are the same as those in section 3.3. On a small data set, the VPSO-FS method does not have advantage in terms of run time because of the number of iterations; for example, it runs more slowly than the two greedy algorithms on the Synthetic Control data set. However, as the number of dimensions increases, the run time of the VPFS algorithm becomes preferable. On the Isolet dataset, the proposed method requires the minimum time of 18.86 seconds for feature subset selection.

Unlike the time complexity of a greedy algorithm, which can be concretely evaluated, the upper bound on the time required for an evolutionary algorithm is difficult to analyze in detail. When the problem space involves inverse matrix operations, the upper bound on the run time is especially difficult to deduce. Therefore, this paper presents a comparison of the run times on specific data. As the size of the data set increases, the run times of CoFS, mRMR, CMFS-*η* and SFS multiplicatively increase. Among the three ranking methods, the run time of CMFS-*η* is the shortest. Between the two subset selection methods, VPFS is much faster than CoFS. The run time of VPFS is mainly affected by the number of bits (feature dimensions), the probability of initializing to 1 bit, and the number of generations. Therefore, as the number of dimensions increases, the run time will increase. However, because the number of iterations is fixed, the overall increase in run time for the ultra-high-dimensional data sets is not significant. When solving for the feature correlation matrix, the VPFS algorithm is relatively time-consuming on large data sets because of the calculation of the eigenvalues. However, during the evolutionary process, the solution time is greatly reduced.

## Conclusion

In this paper, we extend our preliminary study in this paper and propose VPFS algorithm, which transform the data form feature space into a multi-sensor system. On one aspect of feature evaluation, the proposed algorithms achieve the consideration of the combined effect of all features in a subset. On the other aspect of feature search strategy, the VBPSO performs better than traditional BPSO in feature selection. The VPFS algorithm demonstrates good performance in terms of classification accuracy, especially for high-dimensional data. Due to the control of generations, the proposed algorithm has an advantage in time consumption of high dimensional datasets. Different from traditional methods, this paper provides a research perspective for constructing a feature information system. However, the feature correlation matrix is constructed based on mutual information; therefore, when the number of feature dimensions is far greater than the number of instances, the generalization ability of the algorithm will be poor. Science search results of proposed algorithm are not fixed, it is needed for specific practice to get a number of results and determine which is the optimal solution. Future research will focus on feature selection with few training instances for ultra-high-dimensional data. In addition, the trade-off between time consumption and classification accuracy in the searching process should be studied. More than single label in classification, in the next step, we will attempt to extend the correlation information entropy to handle feature selection problems with multi-labels classification.

## Supporting information

S1 Fig Coding scheme of BPSO. (TIF)

S2 Fig S-shaped family of transfer function. (TIF)

S3 Fig V-shaped family of transfer function. (TIF)

S4 Fig General diagram about the proposed algorithm. (TIF)

S5 Fig Comparison with different transfer function on synthetic control. (TIF)

S6 Fig Comparison with different transfer function on mice protein. (TIF)
