# Supplementary figures and images for: Adaptive feature selection using v-shaped binary particle swarm optimization
Source: PLoS One. 2017 Mar 30;12(3):e0173907. doi: 10.1371/journal.pone.0173907 (PMC5373580; doi:10.1371/journal.pone.0173907)

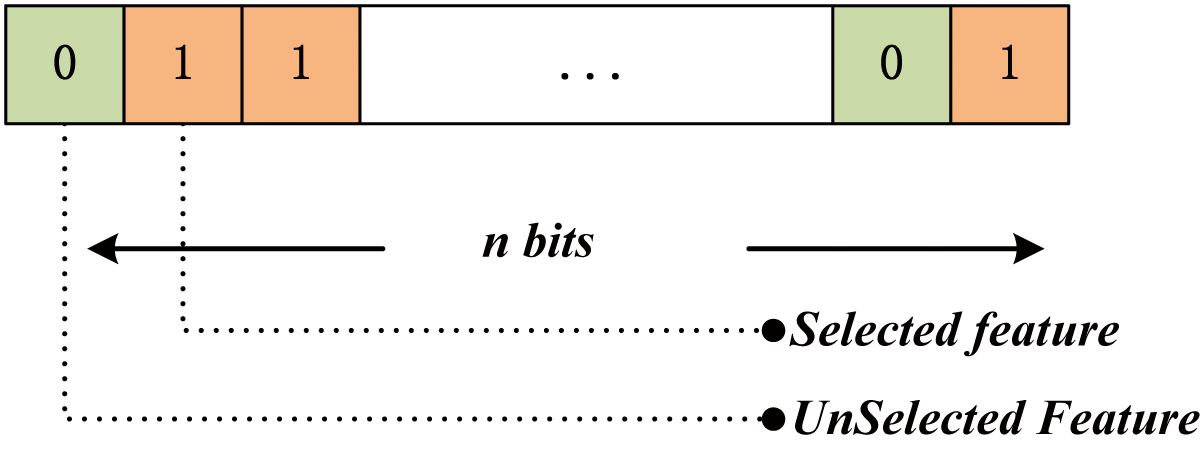

Supplement: S1 Fig — (TIF) [file pone.0173907.s001.tif]

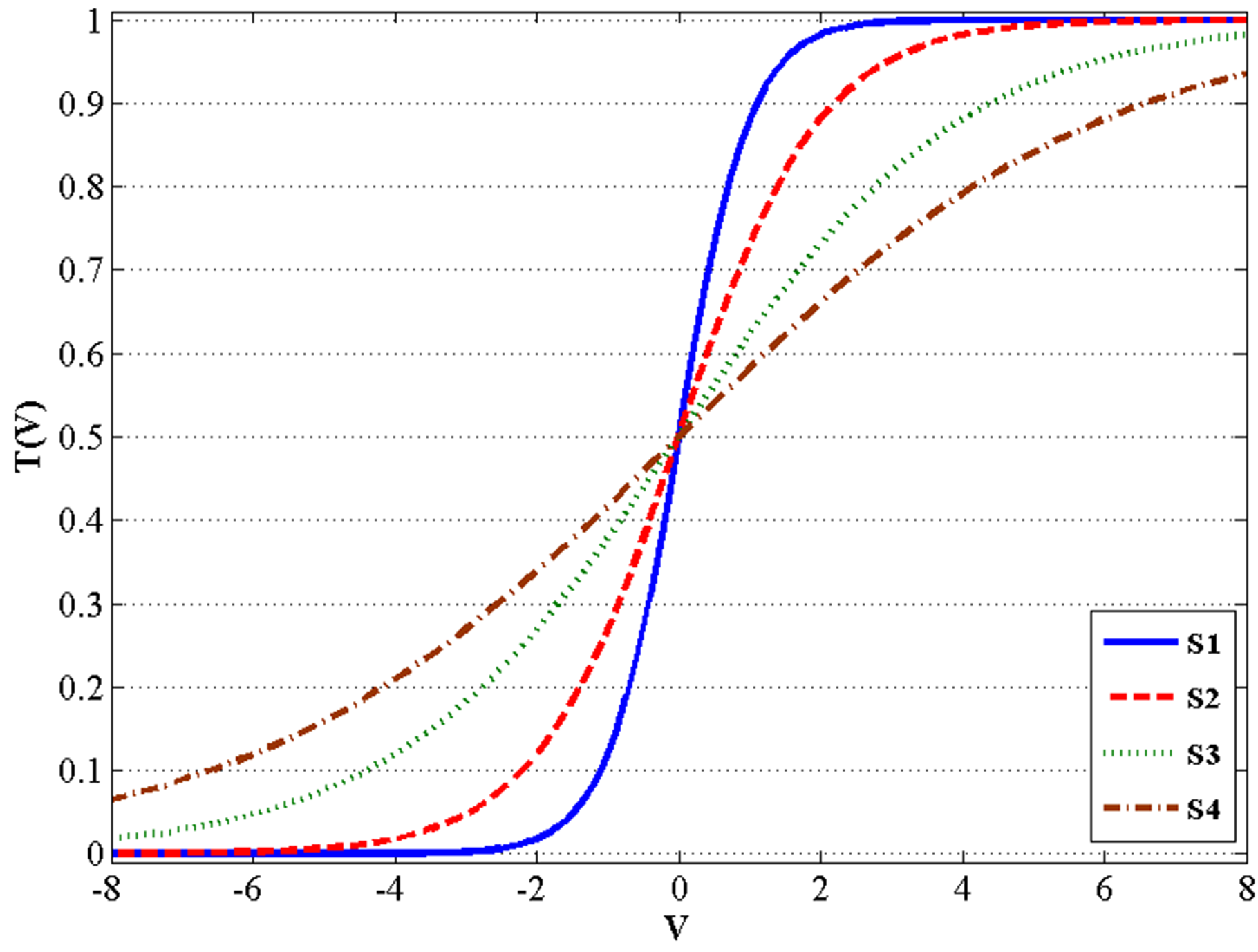

Supplement: S2 Fig — (TIF) [file pone.0173907.s002.tif]

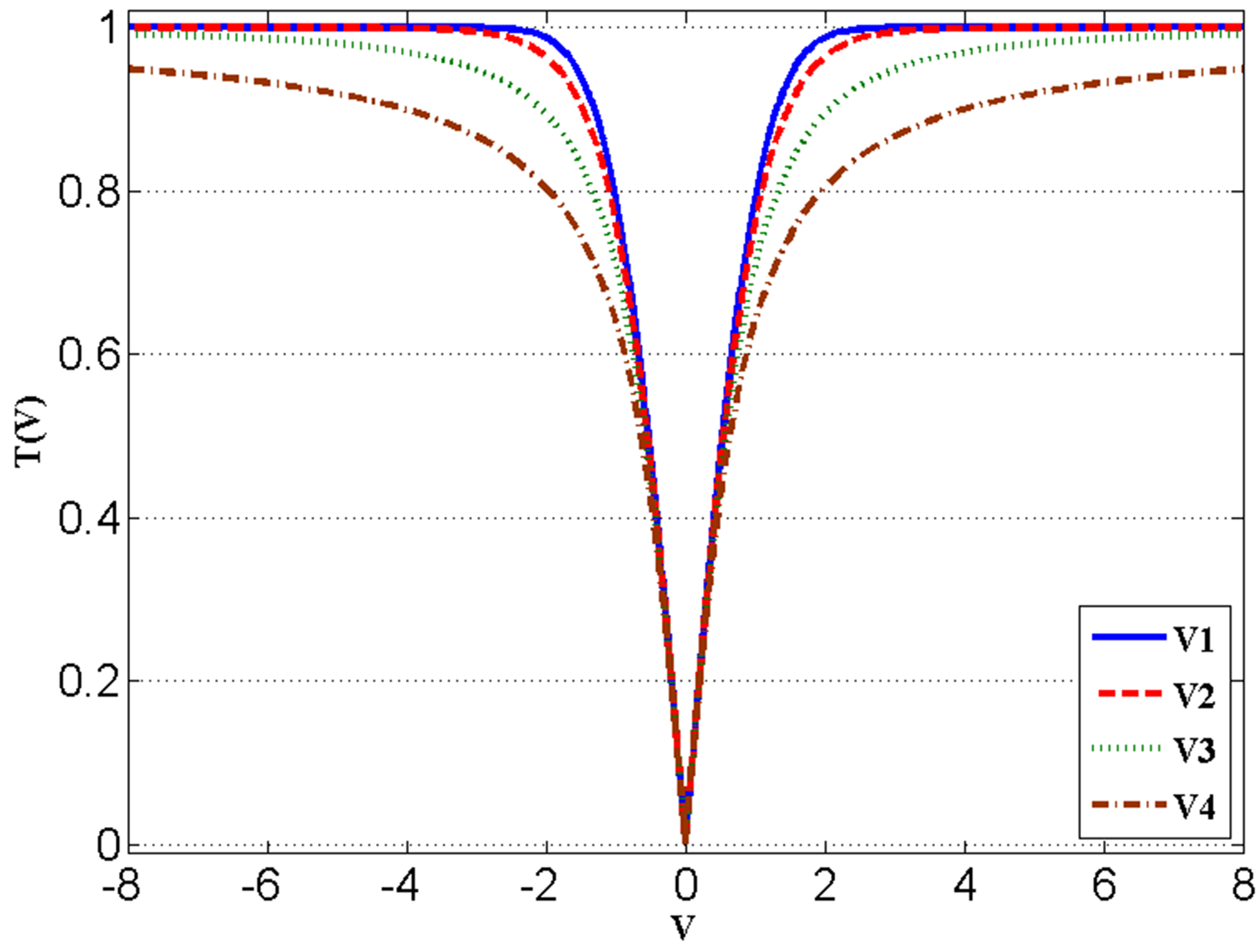

Supplement: S3 Fig — (TIF) [file pone.0173907.s003.tif]

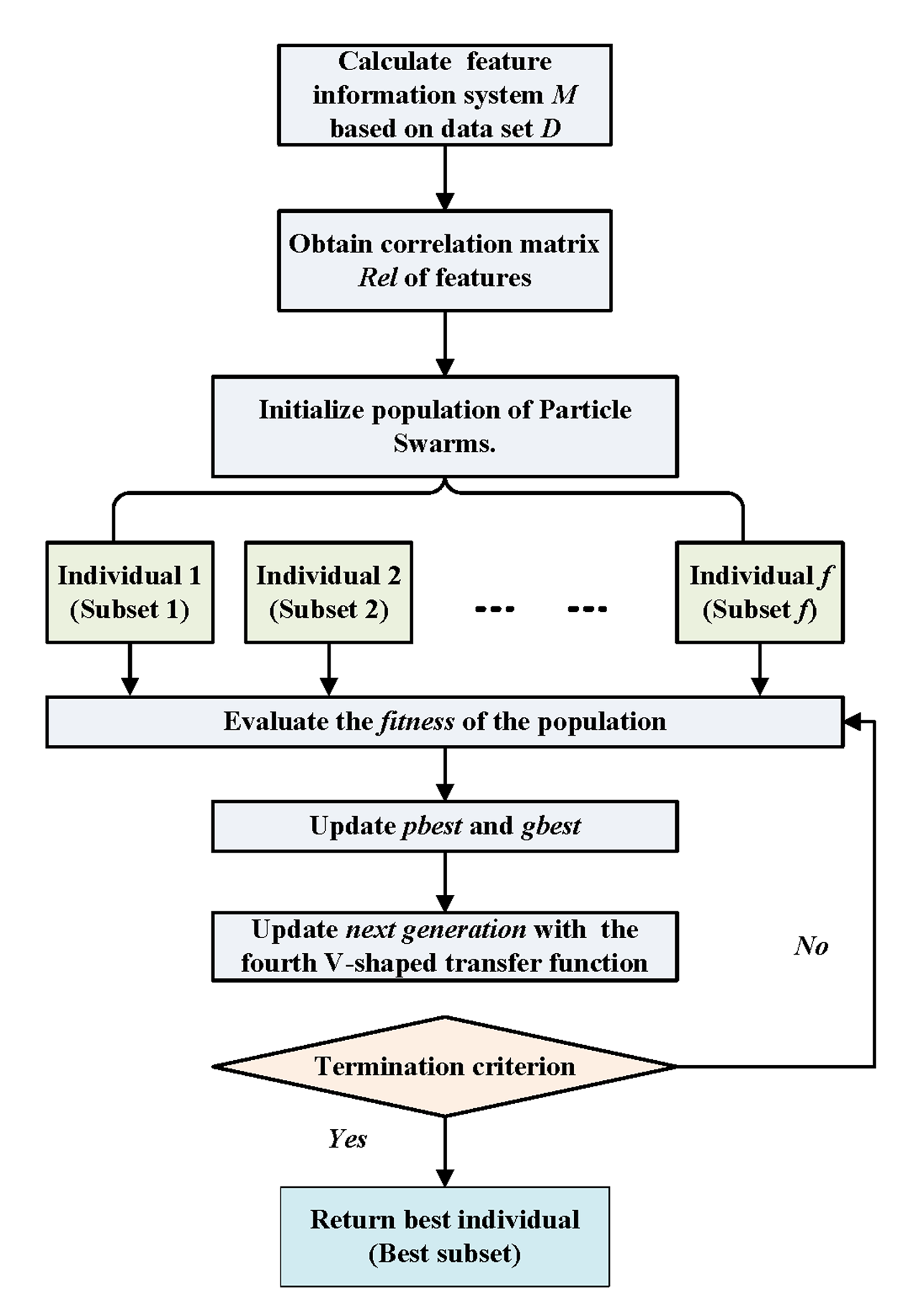

Supplement: S4 Fig — (TIF) [file pone.0173907.s004.tif]

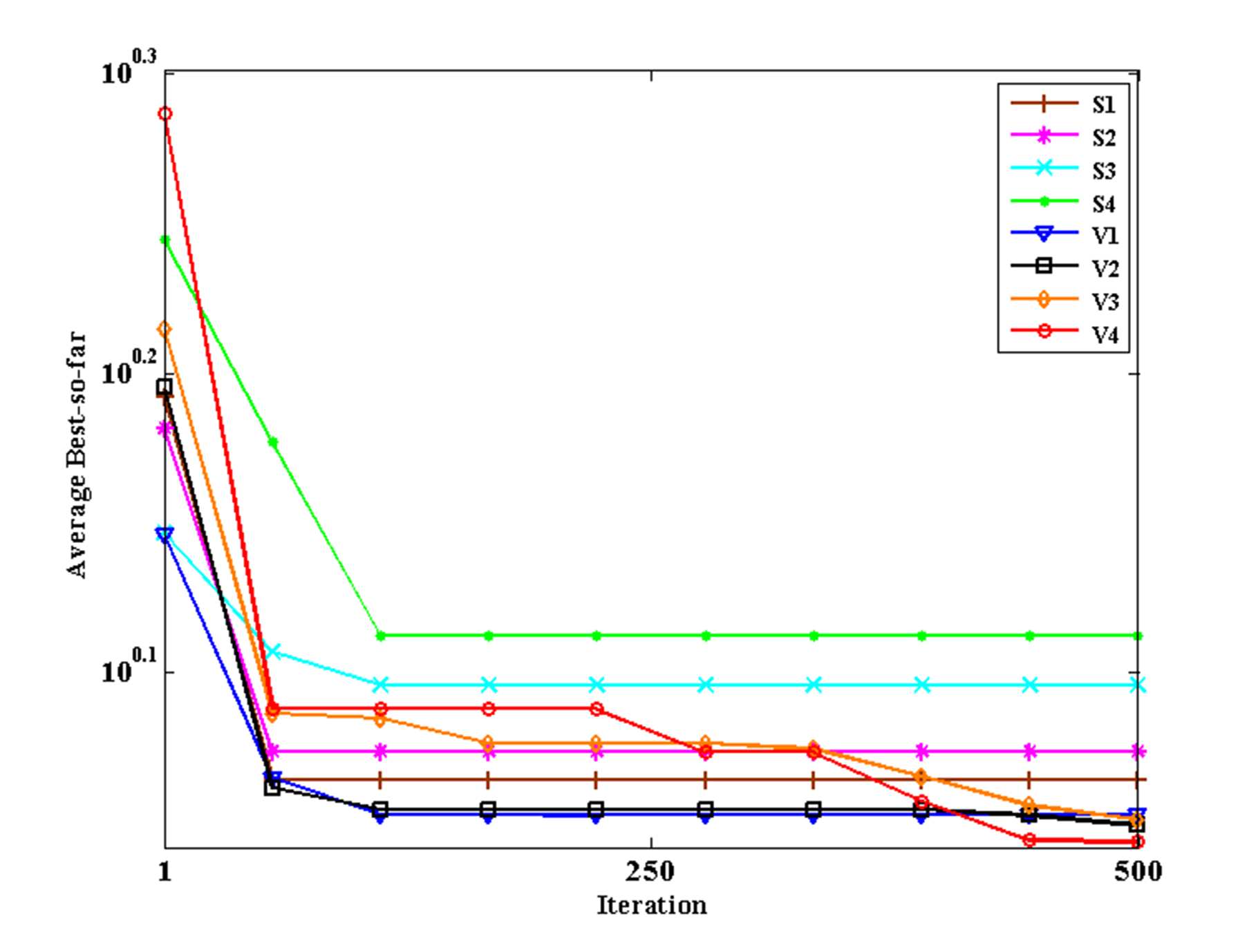

Supplement: S5 Fig — (TIF) [file pone.0173907.s005.tif]

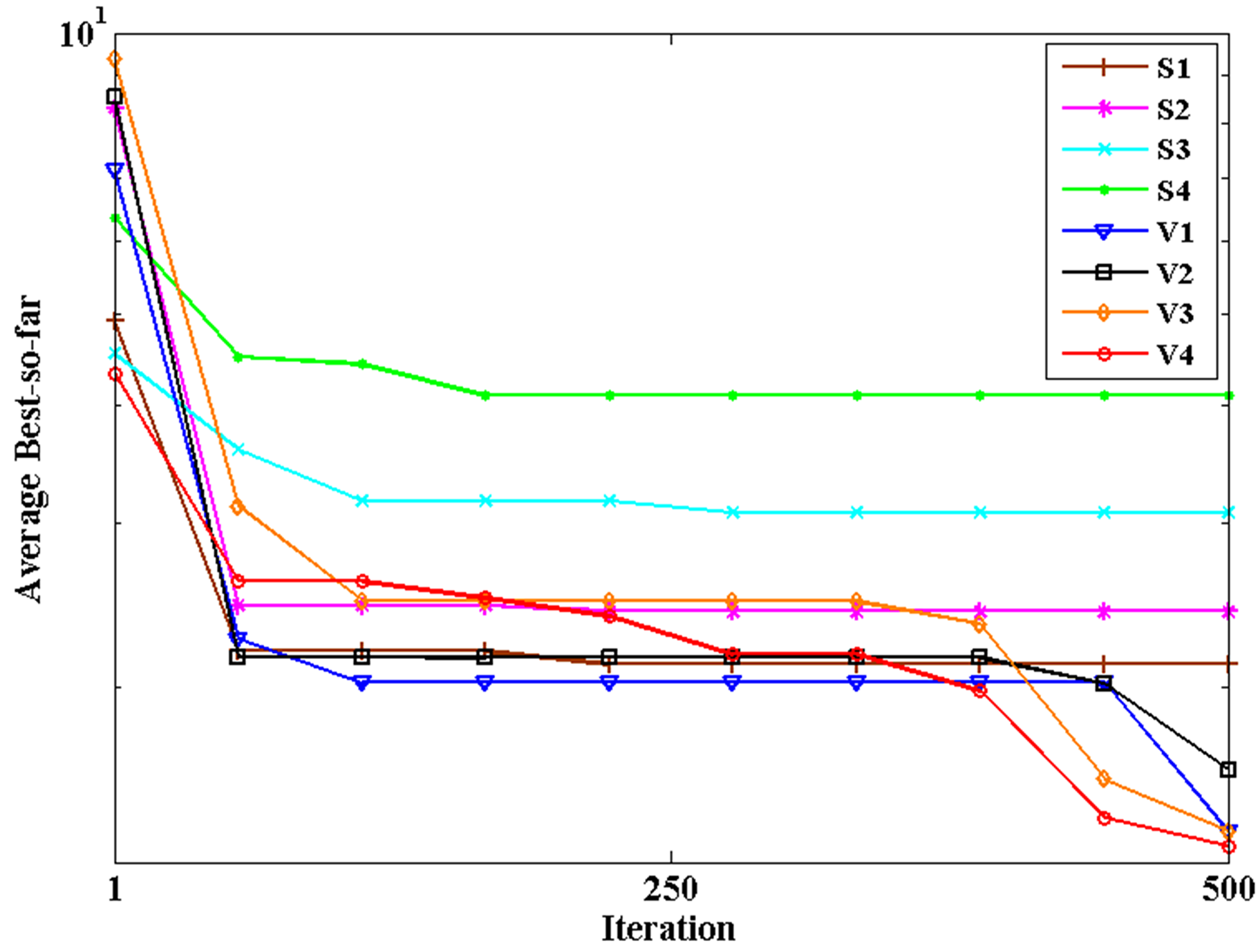

Supplement: S6 Fig — (TIF) [file pone.0173907.s006.tif]
